# Neuro-psycho MMSig: multimodal mechanism enrichment platform for neuropsychiatric disorders

**DOI:** 10.3389/fbinf.2026.1898607

**Published:** 2026-09-23

**Authors:** Vinay Srinivas Bharadhwaj, Karim S. Shalaby, Sathvik Guru Rao, Bruce Schultz, Jiayuan Zhang, Daniel Domingo-Fernández, Martin Hofmann-Apitius, Alpha Tom Kodamullil

**Affiliations:** 1 Department of Bioinformatics, Fraunhofer Institute for Algorithms and Scientific Computing, Sankt Augustin, Germany; 2 Department of Pharmaceutics and Industrial Pharmacy, Faculty of Pharmacy, Ain Shams University, Cairo, Egypt; 3 Department of Biomedical Informatics Köln (BI-K), University of Köln, Köln, Germany; 4 Institut für Informatik, University of Bonn, Bonn, Germany; 5 Enveda Therapeutics, Boulder, CO, United States; 6 Bonn-Aachen International Center for Information Technology (B-IT), University of Bonn, Bonn, Germany; 7 Causality Biomodels, Kinfra Hi-Tech Park, Kalamassery, Kerala, India

**Keywords:** knowledge graphs, mechanism enrichment, network biology, neurological disorders, psychiatric disorders, web application

## Abstract

**Introduction:**

Understanding the pathophysiology of neurodegenerative and psychiatric disorders requires integrating multimodal data.

**Methods:**

We present Neuro-PsychoMMSig, a web-based platform that identifies disease-specific multimodal mechanistic signatures by integrating multi-omics datasets with causal knowledge graphs (KGs). The platform incorporates six curated KGs (Alzheimer's disease, Parkinson’s disease, epilepsy, schizophrenia, bipolar disorder, and type-2 diabetes mellitus) and four network-based algorithms: Clinical Patient Embeddings (CLEP), Candidate Mechanism Perturbation Amplitude (CMPA), Gene Set Enrichment Analysis (GSEA), and CAusal Robust Mapping method in meta-Analysis (CARMA). These tools facilitate qualitative exploration of biomarker interactions and quantitative clinical analysis, including patient stratification, pathway enrichment, and variant fine-mapping.

**Results:**

Application to public Alzheimer’s and schizophrenia datasets identified specific biological dysregulations, including G1/S cell cycle phase transition in Alzheimer’s disease, and clustered distinct patient subgroups.

**Conclusion:**

Neuro-PsychoMMSig offers a locally deployable tool for biomarker discovery, patient subtyping, and mechanistic hypothesis generation.

## 1 Introduction

Neurodegenerative and psychiatric disorders pose significant challenges to neuroscience and clinical medicine, due to their complex pathophysiology and substantial societal and individual burden. Neurodegenerative diseases, such as Alzheimer’s disease and Parkinson’s disease, are multifactorial in nature and require integrative approaches that leverage diverse sources of information and knowledge (Sheikh et al., 2013). Similarly, psychiatric disorders are characterized by multimodal and heterogeneous manifestations, affecting cognition, emotion, behavior, and overall functioning (Admon and Klavir, 2021). The variability in symptoms, disease trajectory, and underlying biological mechanisms complicates efforts to stratify patients into clinically meaningful subgroups. The co-occurrence of neurodegenerative and psychiatric disorders presents significant challenges for both diagnosis and treatment, as these conditions often exhibit overlapping clinical symptoms and share common pathophysiological mechanisms, including neurotransmitter dysregulation and neuroinflammatory processes (Russo et al., 2025). For instance, individuals with neurodegenerative diseases such as Alzheimer’s disease frequently experience psychiatric symptoms including depression and anxiety that may accelerate cognitive decline and diminish quality of life (Mank et al., 2022). In addition, numerous epidemiological studies have reported associations between metabolic and cardiovascular conditions and an increased risk of both neurodegenerative and psychiatric disorders (Cousineau et al., 2024; Gao et al., 2024; Ormazabal et al., 2018; Parlati et al., 2024). This complex interplay highlights the necessity for a comprehensive, interdisciplinary research framework.

In this paper, we present Neuro-PsychoMMSig (Neuro-Psychiatric Multimodal Signature), a platform for identifying disease-specific multimodal mechanistic “signatures” by integrating diverse biological data types and causal knowledge graphs (KGs). These signatures can reveal complex, disease-relevant patterns of dysregulation that are otherwise difficult to capture through unimodal or statistical approaches alone. Neuro-PsychoMMSig is a comprehensive web-based platform integrating four state-of-the-art omics analysis algorithms - CLinical Patient Embeddings (CLEP), Candidate Mechanism Perturbation Amplitude (CMPA), Gene Set Enrichment Analysis (GSEA), and CAusal Robust Mapping method in meta-Analysis (CARMA) - with interactive exploration tools across six curated, disease-specific KGs (Alzheimer’s disease. Parkinson’s disease, epilepsy, schizophrenia, bipolar disorder, and type-2 diabetes mellitus). As each omics analysis algorithm governs different data modality such as GSEA and CLEP corresponding to transcriptomic, CMPA to transcriptomic and proteomic and CARMA to genomic, our platform holistically could be referred to as a multi-omic analysis web application. The platform empowers researchers to uncover complex biological mechanisms, identify biomarkers, generate hypotheses, identify enriched causal mechanisms best suited to the specific biological context or patient subgroup, and support applications such as patient stratification based on their data.

Neuro-PsychoMMSig employs a KG–based approach to integrate multifactorial, heterogeneous information associated with neurodegenerative and psychiatric disorders, alongside multimodal data sources, including genomic and transcriptomic datasets. KGs provide a structured framework for organizing and integrating diverse data types from a variety of sources, including scientific literature, curated databases, and experimental results. Furthermore, KGs can be enriched with semantic annotations, enabling computational reasoning over the integrated data (Neal et al., 2019). Specifically, we utilize the Causal Biological Mechanisms KGs (CBM KGs) (Table 1), which capture causal relationships among biological entities such as genes, proteins, metabolites, and phenotypic outcomes. These relationships span molecular interactions to higher-order disease mechanisms. As such, they serve as a robust foundation for integrating heterogeneous biological knowledge and enabling mechanistic reasoning in complex disease contexts.

**TABLE 1:** Summary statistics on the KGs forming the knowledge base of our tool.

Disease KG	Number of nodes	Number of edges	Number of pathways	Publication
Alzheimer’s disease	5,019	12,455	1,213	Lage-Rupprecht et al. (2022), Domingo-Fernández et al. (2017), Emon et al. (2017), Golriz Khatami et al. (2021), Hoyt et al. (2019), Khanam Irin et al. (2015), Kodamullil et al. (2015), Kodamullil et al. (2017), Naz et al. (2016)
Parkinson’s disease	2,282	4,451	494	Younesi et al. (2015)
Epilepsy	2,390	7,614	642	Hoyt et al. (2018)
Schizophrenia	2,836	5,760	1,934	Bharadhwaj et al. (2023)
Bipolar disorder	2,009	4,586	1,567	Bharadhwaj et al. (2023)
Diabetes mellitus	425	617	182	Karki et al. (2017), Karki et al. (2020)

Neuro-PsychoMMSig has been developed to provide a valuable resource for advancing our understanding of neurological and psychiatric diseases. With the ability to seamlessly query, visualize, and validate any data, including proprietary data, within an intuitive interface, we enable a broad audience of researchers irrespective of their computer science proficiency. To demonstrate the utility of Neuro-PsychoMMSig, we applied it to case studies on Alzheimer’s disease and schizophrenia using publicly available datasets. Our results show that the platform is capable of identifying disease-relevant biological mechanisms (e.g., neurofibrillary tangle assembly and G1/S cell cycle dysregulation in AD) and stratifying patients based on molecular signatures derived from KG embeddings. These findings demonstrate the platform’s potential to support mechanistic hypothesis generation, biomarker discovery, and patient subtyping in complex neurological and psychiatric diseases.

## 2 Methodology

### 2.1 Knowledge graphs

Neuro-PsychoMMSig is pre-installed with six disease-specific Causal Biological Mechanism KGs (CBM KGs) to facilitate comprehensive mechanism-based exploration and analysis for neurological disorders such as Alzheimer’s disease, Parkinson’s disease, and epilepsy, as well as psychiatric conditions including schizophrenia and bipolar disorder. Type-2 diabetes mellitus (T2DM) is also included due to its significant comorbidity and mechanistic overlap with schizophrenia and bipolar disorder (Bharadhwaj et al., 2023). The CBM KGs were manually curated and encoded using Biological Expression Language (BEL) (Slater, 2014), providing a rich semantic framework capable of capturing detailed causal interactions among multimodal entities such as genes, proteins, metabolites, biological processes, phenotypes, and chemicals. Each KG is highly granular, comprising thousands of causal relationships (edges) and biological entities (nodes), enabling precise mechanistic reasoning (see Table 1 for details). A schema diagram illustrating the multimodal and granular nature of these KGs, including node and relationship diversity, is presented in Figure 1, and the detailed attributes of these nodes and edges are provided in Supplementary Tables S1 and S2. Furthermore, users can incorporate their own KGs into the Neo4j database with minimal preprocessing.

**FIGURE 1:**
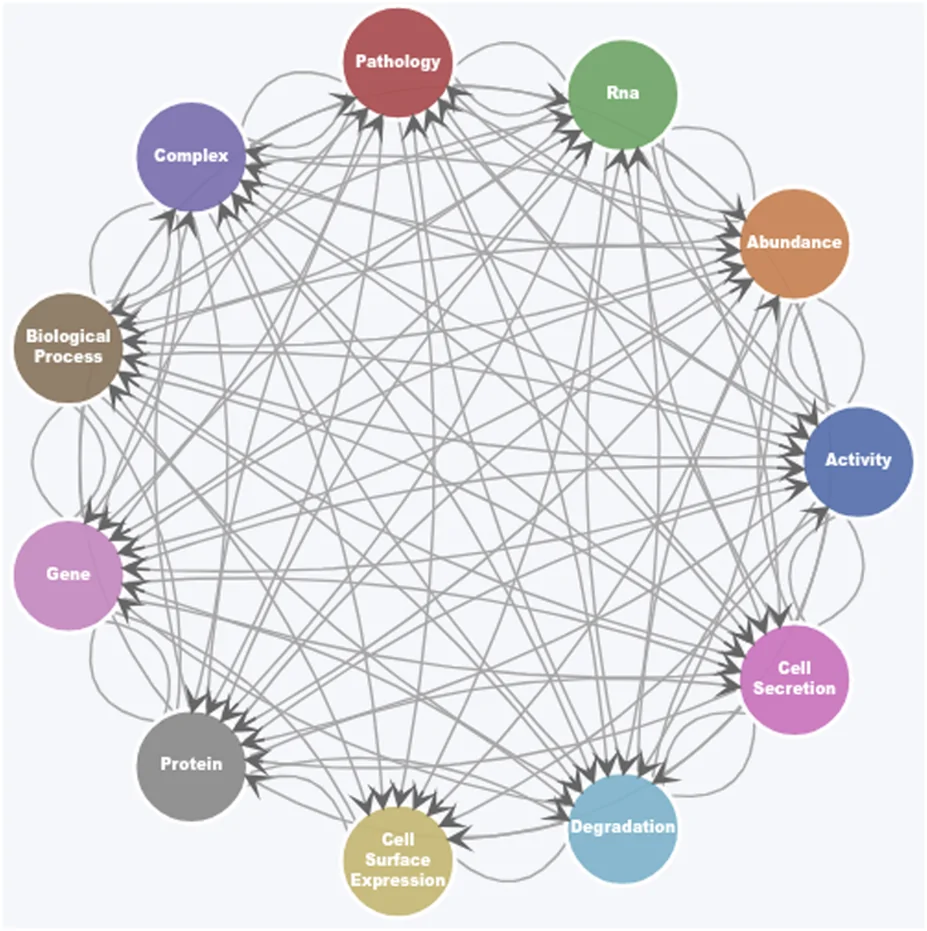
Schema diagram illustrating the diverse node types (e.g., genes, proteins, biological processes, metabolites) and causal relationships present between them in Neuro-PsychoMMSig’s CBM KGs.

### 2.2 Implementation

Neuro-PsychoMMSig uses a Neo4j Community Edition database that is hosted in a Docker container. The Neo4j instance only allows the creation of 1 database with no user permissions. To combat this, we have merged all the disease KGs into a single database and created an edge attribute called “disease” to allow the user to distinguish different disease KGs. By default, write permissions on the database files are disabled to ensure that server administrators are able to control database content and versioning. The web server is built using the Python-based Django web development framework (https://www.djangoproject.com/). Django’s Model-View-Template (MVT) architecture was employed to manage the backend, while its built-in tools were leveraged for handling authentication, form processing, and database interactions. The KG database interactions were configured using the neomodel (https://neomodel.readthedocs.io/en/latest/) Python library, which provides the object graph mapper (OGM) for Django.

The algorithms are implemented using multiple Python libraries such as pandas (https://pandas.pydata.org/), networkx (https://networkx.org/), PyKEEN (https://pykeen.readthedocs.io/en/stable/), and PyTorch Geometric (Fey et al., 2017/2025; Fey and Lenssen, 2019). To allow for a more interactive experience, we have also implemented an asynchronous task execution using RabbitMQ (https://www.rabbitmq.com) worker containers and Celery (https://www.celeryproject.org) Python library to execute the algorithms on the worker containers. The front-end of Neuro-PsychoMMSig comprises several interactive visualizations implemented using a collection of powerful JavaScript libraries, including jQuery (https://jquery.com), D3. js (https://d3js.org/) and DataTables (https://datatables.net/). Furthermore, Neuro-PsychoMMSig relies on Bootstrap 5 (https://getbootstrap.com/) for the website’s main design. The web application is containerized using Docker for reproducibility purposes and easy deployment.

### 2.3 Implemented algorithms

Neuro-PsychoMMSig consists of two main analysis methods, the first one being an explorative analysis, which is a way to interact with KGs (see Section 2.3.1). Meanwhile, the second method, clinical analysis, allows the user to carry out network-based algorithms on -omics datasets using KGs as prior knowledge. Our platform includes four algorithms, with the ability for a developer to create more KG- or dataset-based algorithms to deploy in their version of Neuro-PsychoMMSig (see Section 2.3.2).

#### 2.3.1 Explorative analysis

Neuro-PsychoMMSig offers qualitative exploration capabilities, allowing users to interactively query the CBM KGs using biomarkers. This interactive visualization enables exploration of subgraphs, identification of relationships’ provenance, and insights into the structure and connectivity of disease mechanisms. Visualization uses D3. js for dynamic graph rendering. Graph-based exploration methods available include subgraph extraction and analysis of local graph neighborhoods to explore specific biological contexts. For an exploratory analysis, a user can select specific biomarkers, such as genes, proteins, subgraphs (pathways), biological processes (nodes in the KG), or diseases/symptoms across multiple disease-specific KGs (Figure 2). For instance, as shown in Supplementary Figure S1, a user can simultaneously query IL6 gene interactions in schizophrenia, the Alzheimer’s disease subgraph, and the epilepsy KG, focusing on seizures. Additionally, users can refine their searches by adjusting parameters like the search depth or narrowing the search by visualizing the shortest path between all the entered terms, enabling targeted insights into mechanistic relationships across diseases. This flexibility supports hypothesis generation and helps uncover common or distinct and specific molecular pathways relevant to multiple disorders.

**FIGURE 2:**
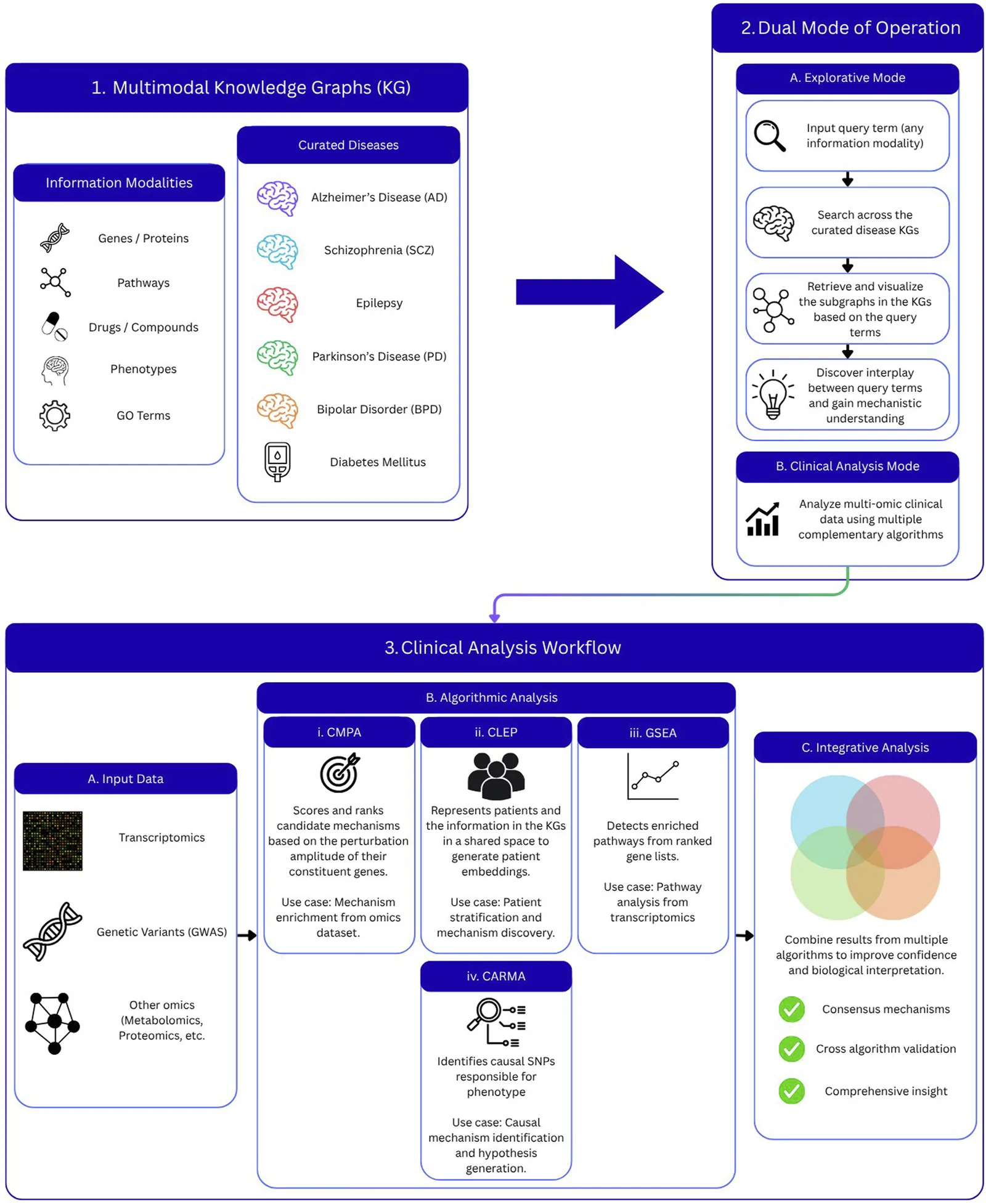
Overview of the Neuro-PsychoMMSig platform and analysis workflows. The Neuro-PsychoMMSig web application consists of three components. (1) Multimodal knowledge graphs, which are disease-specific knowledge graphs for Alzheimer’s disease, Parkinson’s disease, schizophrenia, bipolar disorder, epilepsy and diabetes mellitus. (2) Dual mode of operation: users can either perform “Explorative Analysis”, which allows for interactive querying and visualization of disease-specific knowledge graphs to investigate mechanistic relationships, or “Clinical Analysis”, which supports analysis of patient-derived multi-omics datasets using complementary computational approaches. (3) Clinical analysis workflow analyzes clinical data using four complementary algorithms, CMPA for mechanism enrichment, CLEP for patient representation learning and stratification, GSEA for pathway enrichment analysis and CARMA for causal mechanism identification. The integration of results across algorithms provides complementary biological evidence, improving confidence in mechanistic interpretation and hypothesis generation.

#### 2.3.2 Clinical analysis

Neuro-PsychoMMSig provides comprehensive data analysis capabilities, enabling users to perform network-based mechanistic analyses of multi-omics datasets integrated with disease-specific KGs. This clinical analysis mode incorporates four advanced computational algorithms (Figure 2), CLinical Embeddings with Patient data (CLEP) (Bharadhwaj et al., 2021), Candidate Mechanism Perturbation Amplitude (CMPA) (Karki et al., 2019), Gene Set Enrichment Analysis (GSEA) (Subramanian et al., 2005), and CAusal Robust Mapping method in meta-Analysis (CARMA) (Yang et al., 2023) each tailored for specific biological data modalities, including gene expression and genome-wide association studies (GWAS). These algorithms utilize curated domain-specific KGs as prior knowledge, thereby enhancing the interpretability and relevance of analytical results. Users can upload their omics datasets, and analyses are executed asynchronously, providing interactive visualizations of results with detailed provenance and contextual biological insights (Supplementary Figure S2A, B).

##### 2.3.2.1 Clinical embeddings with patient-level data (CLEP)

In the CLEP (Bharadhwaj et al., 2021) algorithm, the KG acts as *a priori* information to boost the quality of the gene expression dataset using KG embedding models (Supplementary Table S3). In this publication, we use the same strategy to boost the clustering efficiency of gene expression data for patient subgroup identification. In CLEP, the patient embeddings were generated by connecting the patients to the genes in the graph based on the gene expression data. Because generating patient embeddings can take several hours, we have changed the implementation in the website by first generating node embeddings for all the genes in the KG using a heterogeneous graph transformer (Hu et al., 2020), and then the patient embedding is generated by combining the node embedding of the genes in the dataset.

##### 2.3.2.2 Candidate Mechanism Perturbation Amplitude (CMPA)

Candidate mechanism perturbation amplitude (CMPA) (Karki et al., 2019) is a quantitative measure to evaluate the extent to which a specific candidate mechanism (e.g., a gene, protein, or pathway) can alter the behavior of a biological system when perturbed. This metric is typically applied in the context of hypothesis testing or model validation to assess the significance of a proposed mechanism in driving observed changes or phenotypic outcomes (Supplementary Table S3). The CMPA metric is calculated by applying the gene expression values to the nodes in a graph and propagating the scores throughout the graph by multiplying the expression scores with the edge attribute. This results in an overall metric for the graph which determines the amplitude and direction of the perturbation. However, this calculation is computationally intensive for a larger disease KG, hence in our implementation of CMPA, we have opted to calculate the perturbation of each biological process node in the KGs by creating an ego graph of neighbourhood rank 2. This creates a graph centered around each biological process and calculates the CMPA metric for the biological process using genes closest to it, instead of calculating it for the whole KG.

##### 2.3.2.3 Gene Set Enrichment Analysis (GSEA)

Gene set enrichment analysis (GSEA) (Subramanian et al., 2005) is a computational method used to determine whether predefined sets of genes (e.g., pathways, functional categories, or other biologically meaningful groups) show statistically significant, coordinated expression changes under specific experimental conditions (Supplementary Table S3). Our implementation of GSEA does not utilise the KG but instead uses pathway databases as the source or the gene sets. This enables us the users to analyse their datasets with a mature information data source. GSEA operates by ranking all genes based on their correlation with a particular phenotype or experimental condition and assessing whether members of a gene set are overrepresented at the extremes of this ranked list. This approach is particularly effective for detecting subtle but coordinated changes in gene expression that might be overlooked when analyzing genes individually. This is used to identify the dysregulated pathways of a phenotype, such as patients diagnosed with schizophrenia and patients without such a diagnosis.

##### 2.3.2.4 CAusal Robust Mapping method in meta-analysis (CARMA)

Lastly, we have also implemented CARMA (Yang et al., 2023), which is a Bayesian model for fine mapping GWAS datasets and is used for the identification of putative causal single nucleotide polymorphisms (SNPs) of a sample. Fine-mapping methods leverage probabilistic models to compute posterior probabilities for each variant being causal, often incorporating prior knowledge from epigenomic datasets, transcription factor binding sites, and chromatin accessibility profiles. This approach has been instrumental in bridging the gap between GWAS signals and functional biology, enabling researchers to focus on specific variants for downstream experimental validation (Supplementary Table S3). In order to use the CARMA library, we have created R scripts that generate the pairwise linkage disequilibrium (LD) matrix and generate functional annotations using the 1,000 Genome Project using the polyfun R package (Weissbrod et al., 2020). This allows us to identify putatively causal SNPs that may underlie a phenotype. The CARMA algorithm could be used to carry out variant effect prediction using a foundational model such as AlphaGenome (Avsec et al., 2026)

### 2.4 Installation

Neuro-PsychoMMSig can be accessed publicly at https://mmsig.scai.fraunhofer.de, however in the case of larger datasets or cases where the compute capacity of this public server may be insufficient, depending on the type of analysis, or due to data privacy regulations, users can install and use Neuro-PsychoMMSig on their own network. The easiest method of deployment is to install Docker and deploy the Docker container, which will install the required components and run the web application. Detailed instructions are provided on https://github.com/Fraunhofer-SCAI-Applied-Semantics/MMSig.

### 2.5 Runtime considerations

Computation time is dependent on the type of analysis, size of the datasets, as well as the device specifications. Algorithms such as GSEA and CMPA are relatively light packages and can be computed in a short period of time. CARMA analysis takes up to 1 h for each analysis run, and the CLEP analysis is carried out in less than 10 min. Finally, our software includes an optional notification system in which the deployment can be configured to send an email to the user via SMTP about individual job updates.

### 2.6 Case scenario

#### 2.6.1 Alzheimer’s disease

For benchmarking our software, we utilized the data from Alzheimer’s Disease Neuroimaging Initiative (ADNI) database (adni.loni.usc.edu) (Mueller et al., 2005). The ADNI was launched in 2003 as a public-private partnership, led by Principal Investigator Michael W. Weiner, MD. The primary goal of ADNI has been to test whether serial magnetic resonance imaging (MRI), positron emission tomography (PET), other biological markers, and clinical and neuropsychological assessment can be combined to measure the progression of mild cognitive impairment (MCI) and early Alzheimer’s disease (AD). The dataset, which is already preprocessed, includes 260 cognitively healthy control participants, 215 patients with early mild cognitive impairment, 225 patients with late mild cognitive impairment, and 44 patients with Alzheimer’s disease. For the binary classification task, the cognitively impaired patients (n = 484) were grouped into a single class, while preprocessed gene expression data [Robust Multichip Average (RMA) normalized] was used as a baseline for benchmarking CLEP. To incorporate the patients into the KG, the expression of genes with multiple transcripts was considered individually. We used blood plasma transcriptomic and genome-wide association study (GWAS) data collected in ADNI [details in Saykin et al., 2015 and http://adni.loni.usc.edu]. The processing of the transcriptomic data for CLEP, CMPA, and GSEA analysis was performed in a similar manner as described in our CLEP publication (Bharadhwaj et al., 2021). Using the CLEP embeddings, we carried out subgroup identification of ADNI patients and benchmarked its performance by comparing it with the similarity network fusion algorithm used by Li H. T. et al. (2021). The Leiden graph clustering (Traag et al., 2019) was carried out on a connectivity network generated using the “kneighbors graph” function in the scikit-learn Python library. Finally, we also used the ADNI GWAS dataset to carry out CARMA analysis by using the summary statistic generated by Wightman et al. (2021), and mapping functional annotations to each SNP in the GWAS dataset (Gazal et al., 2018; Weissbrod et al., 2020).

#### 2.6.2 Age-stratified Alzheimer’s disease analysis

We utilized the GSE33000 (Narayanan et al., 2014) gene expression dataset, which comprises *postmortem* prefrontal cortex samples from 624 individuals, including both demented and non-demented control subjects. To assess age-related gene expression changes, we stratified patients into four age bins: 50–69, 70–79, 80–89, and 90–100 years. Differential expression analysis was conducted using the Geo2R tool (GEO2R, 2025) to compute log fold change (logFC) values for each age group. The resulting gene expression profiles were further analyzed using CMPA, CLEP, and GSEA. These analyses allowed us to identify differentially co-expressed gene pairs, disrupted molecular pathways, and key regulatory networks associated with neurodegenerative disease pathology across different age groups.

#### 2.6.3 Schizophrenia disease

We also benchmarked our platform for schizophrenia using 2 gene expression datasets: GSE53987 (Lanz et al., 2019) and GSE21138 (Narayan et al., 2008; Tang et al., 2011, Tang et al., 2012). The GSE53987 dataset comprises the microarray profiles from schizophrenia, bipolar disorder, and major depressive disorder. The gene expression data were collected from three different brain regions (hippocampus, prefrontal cortex, and striatum) of post-mortem brain samples. We carried out the CMPA, using the differential gene expression analysis, and the GSEA and CLEP analysis using 48 schizophrenia samples and 55 unaffected samples. The GSE21138 dataset consists of the microarray gene expression profiles from the prefrontal cortex samples of 30 *postmortem* schizophrenia brains and 29 age- and sex-matched controls. Similar to GSE53987, the GSE21138 dataset was analyzed using CMPA, GSEA, and CLEP algorithms.

## 3 Results and discussion

We have created a web platform, Neuro-PsychoMMSig, that enables intuitive exploration and navigation through its graph database by generating subgraphs based on biomarkers such as genes, proteins, and biological processes across all diseases included in the platform (Figure 2). Additionally, Neuro-PsychoMMSig supports data analyses using KG algorithms, such as CLEP, CARMA, GSEA, and CMPA on patient-level datasets to identify dataset-specific results and also to compare and contrast the overlapping results of the different algorithms. The clinical data analysis requires user registration, whereas the explorative functionality is free to use. A quickstart guide on the website summarizes the various functionalities (Supplementary Figure S3A).

### 3.1 Explorative analysis

Explorative analysis allows users to query the database using individual biomarkers for specific diseases of interest. Users can combine multiple query terms to identify shared interactions across different disease contexts, enhancing the discovery of common and unique biological mechanisms. This exploratory functionality does not require user registration. Once a query is submitted, the Neo4j database generates a subgraph illustrating all connections to and from the selected biomarker(s).

Exploratory analysis helps researchers identify both shared and unique biomarkers for different diseases, highlighting inter-disease relationships, as shown in Figure 3A. Users can simultaneously query multiple KGs, e.g., Alzheimer’s and Parkinson’s disease KGs, to illustrate overlapping interactions, or query disease-specific biomarkers to identify unique metabolic pathways and disease-specific mechanisms, such as protein and biological process interactions relevant to the disease. The KG visualization page further enriches exploration by allowing detailed inspection of individual edges within the graph, as shown in Figure 3B. Users can click on any edge to access provenance details, including supporting publications. This feature ensures transparency, enabling validation and a deeper understanding of biological interactions identified through the platform.

**FIGURE 3:**
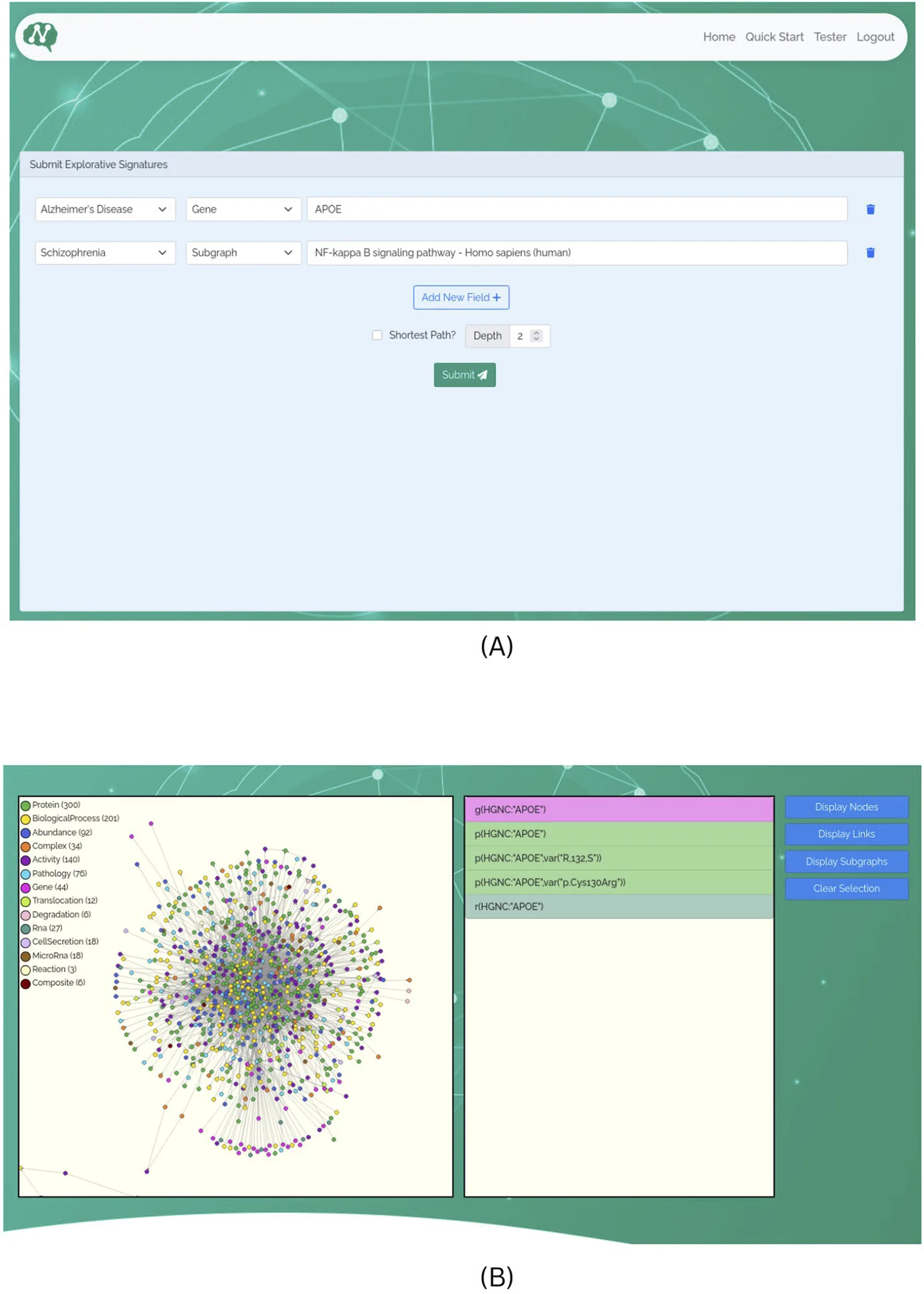
**(A)** Explorative Analysis. The web UI for exploring the shared context between APOE in Alzheimer’s disease KG and NF-kappa β signalling pathway in schizophrenia KG. **(B)** Visualization of explorative analysis. This is the resultant subgraph of the queried APOE node and NF-kappa β signalling pathway.

### 3.2 Clinical analysis

Clinical analysis, in contrast, allows one to perform network-based analyses in the context of these disease-specific KGs using either the provided or uploaded datasets. Once registered, users can select an analysis method, upload necessary datasets, and initiate computational workflows. The format requirements for each file differs depending on the algorithm used, for example, CLEP, GSEA and CMPA algorithms require a transcriptomics data file and a design matrix file. The required format for each algorithm is described on the website as well as on the GitHub page. For carrying out the CARMA analysis, the user needs to submit the LD matrix of the GWAS dataset along with optional functional annotations. The CARMA publication recommends the augmentation of functional annotation with the GWAS dataset for the accuracy of the fine mapping process. Once the analysis type has been chosen and the relevant dataset has been uploaded, the user can submit the job, which is then executed on one of the workers. Once the job is finished processing, this can range anywhere from 1 min to 24 h, depending on the analysis selected. An email is dispatched notifying the user upon job completion with a URL to the results page (Supplementary Figure S4). The job can also be accessed using the accounts tab, where all the jobs submitted by the user are listed.

In this section we outline the results of the two AD case studies with ADNI dataset where we carry out all the 4 implemented algorithms on the genetic variant data and the transcriptomic data available in the ADNI web portal. The second case study follows an age subgroup dataset, GSE33000, where we utilize transcriptomic data to stratify patients and identify key mechanistic patterns between the age groups. We also have carried out a case study on two schizophrenia datasets, GSE53987 and GSE21138 to get a better mechanistic understanding of schizophrenia with the transcriptomic data.

#### 3.2.1 AD case study

In this study, we utilized the Alzheimer’s Disease Neuroimaging Initiative (ADNI) dataset as the primary source of genetic and biological data. The CMPA algorithm was applied to calculate the perturbation impact of genes associated with Alzheimer’s disease (AD) on the Alzheimer’s disease KG. The application of the CMPA algorithm on disease-specific graphs enabled us to quantify how AD gene data perturbed key biological processes within the network. In the Alzheimer’s disease-specific graph, we observed a dysregulation of GO biological processes such as cell growth (GO:0016049), cell cycle G1/S phase transition (GO:0044843), stress response to copper ion (GO:1990169) and neurofibrillary tangle assembly (GO:1902988). These findings are in line with previous findings by researchers including the G1/S cell phase transition (Bonda et al., 2010). Bonda et al. notes the significance of cell cycle alteration in AD as the cell cycle is mainly controlled by cyclins and cyclin-dependent kinases (CDKs). And the neurons of AD patients exhibit significantly elevated levels in CDKs. Similarly, the role of copper ions has also been associated with AD and is linked to the development of AD (Ejaz et al., 2020; Li et al., 2025). Multivalent metal ions such as copper have been reported to be at higher levels in AD patients, and mouse models have also shown evidence of chronic copper exposure leading to neurodegeneration (Lim et al., 2020). Furthermore, GSEA analysis also revealed the dysregulation of “Caspase activation *via* Death Receptors in the presence of ligand” and “Caspase activation via extrinsic apoptotic signalling pathway”. Both of these pathways govern apoptosis. CMPA results supplement the GSEA results as it provides an insight into the mechanism of action by revealing the link to copper ion build up. Li et al. (2025) mentions that a dysregulation in the copper homeostasis could lead to the inhibition of lipid and protein lipoylation modifications, which could lead to mitochondrial dysfunction and eventually cell death.

For the validation of the new CLEP algorithm using the HGT transformer as detailed in the methods Section 2.3.2.1, we benchmarked the analysis by comparing the cluster assignment identified by H.-T. Li et al. with the Leiden-based clustering results of the patient embeddings. The Leiden algorithm is a community detection algorithm that clusters nodes in a graph based on their similarity. For carrying out the Leiden algorithm (Traag et al., 2019) on our embedding data, we generated a connectivity matrix of patient embeddings using scikit-learn. The Leiden clustering identified 2 clusters, similar to the H.-T. Li et al. publication, and had a silhouette score of 0.48 and a modularity of 0.46. The clustering metrics were then calculated to find the similarity of our clustering results and the cluster assignments depicted in the publication. These metrics can be seen in Table 2 below. The metrics indicate 45% similarity in the cluster assignments between the similarity network fusion approach and our patient embedding approach for subtype identification.

**TABLE 2:** Clustering metrics results of the Leiden clustering analysis on the ADNI data.

Metric	Score
Adjusted Rand Index (ARI)	0.4581702073315447
Normalized mutual information (NMI)	0.3850293203217746
Completeness	0.38979484998127323
Homogeneity	0.38037890750678155
Fowlkes mallows score	0.7316275149347274

Furthermore, using this new CLEP analysis, we analysed all the patients’ gene expression data to identify patient subtypes in AD and MCI subgroups, however the inclusion criteria for the subtypes need to be assessed further. Figure 4 depicts a normalized PCA representation of the subtypes.

**FIGURE 4:**
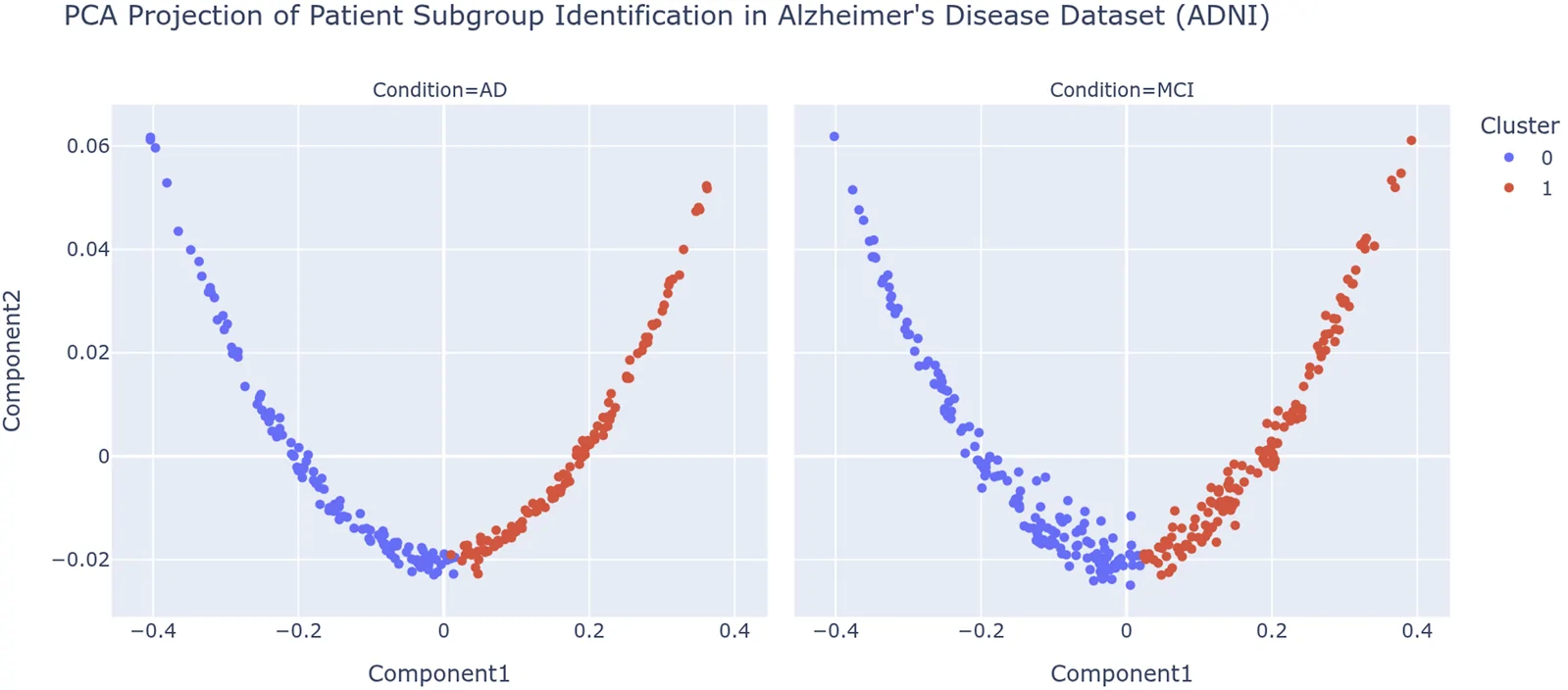
CLEP Analysis of ADNI. Results of CLEP analysis on the ADNI dataset, which are then visualised using a PCA projection of the CLEP embeddings.

Finally, using ADNI’s GWAS dataset, we were able to carry out fine mapping using CARMA to identify the putatively causal SNPs. However, the CARMA results were not able to identify any significant loci/region of SNPs related to AD, but it was able to statistically determine that rs9267810, a SNP in the intron region of Palmitoyl-Protein Thioesterase 2 (PPT2) gene on chromosome 6, has 97.5% probability to be a causal SNP. Although the SNP itself is not well known or researched in the context of AD, the PPT2 gene has been associated with AD in mice knockout studies (Gupta et al., 2001) as well as the mechanism of action for Palmitoyl-Protein Thioesterase 1 (PPT1), the sister protein of PPT2, has been identified and linked to AD (Cho and Park, 2016).

#### 3.2.2 AD sample stratification by age

For the GSE33000 dataset, the CMPA analysis was carried out to identify the pattern over different age groups of AD patients. CMPA was able to identify a positive trend in the dysregulation of biological processes such as neuron differentiation (GO:0030182), amyloid fibril formation (GO:1990000), cell cycle G1/S phase transition (GO:0044843). This trend represents the increase in dysregulation as an individual ages. However, it is to be noted that the GSE33000 dataset is not a time series dataset but instead groups patients into age bins (50–69, 70–79, 80–89, and 90–100 years). Similarly, a negative trend was also noted in biological processes such as regulation of MAPK cascade (GO:0043408), neutrophil aggregation (GO:0070488) and regulation of ER-associated ubiquitin-dependent protein catabolic process (GO:1903069). The upward trend in the dysregulation of neuron differentiation and amyloid fibril formation is expected in AD patients and the dysregulation of the G1/S phase transition process is in line with the results from ADNI data analysis. Also, the role of neutrophil adhesion has been associated with the impairment of memory in mice models (Cruz Hernández et al., 2019). It is also known that the downward trend in the dysregulation of positive regulation of ERAD pathway has been linked to neuronal degeneration (Zhu et al., 2017). The GSEA analysis across all the age groups showed the upregulation of genes involved in the cristae formation (R-HSA-8949613) and prior publications have identified that the amyloid oligomers might induce the ion formation in the mitochondria (Khalifat et al., 2012). The GSEA analysis also showed the downregulation of the genes involved in Interleukin (IL)-27 signaling (R-HSA-9020956) which has been linked to neuroprotective and neuroinflammatory processes (Nortey et al., 2022). Figure 5 presents CLEP-based clustering results, clearly showing distinct patient clusters across age groups.

**FIGURE 5:**
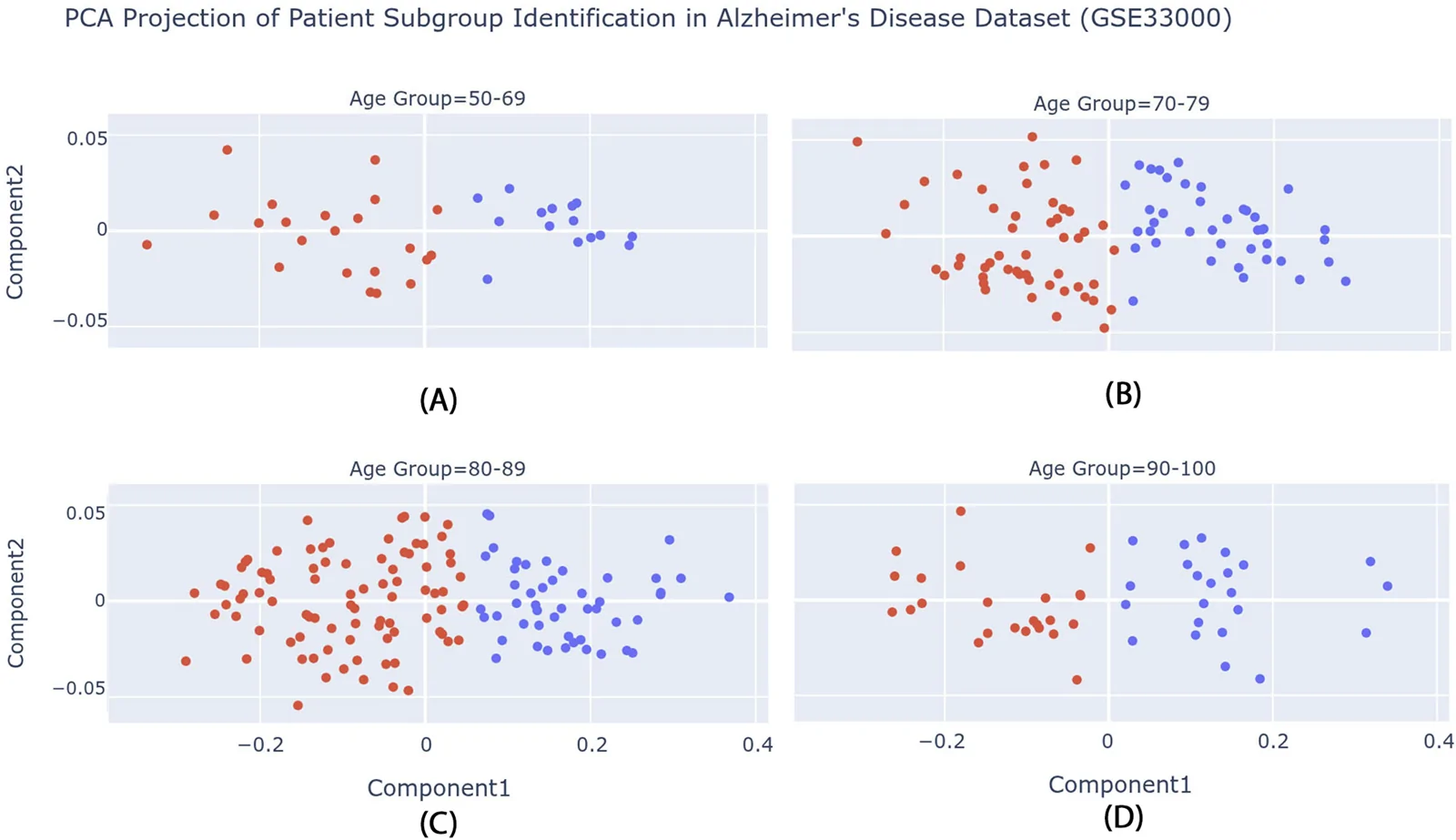
CLEP Analysis of GSE33000. Clustering of the CLEP embeddings generated for GSE3300 **(a)** age groups 50–69, **(b)** age groups 70–79, **(c)** age groups 80–89, **(d)** age groups 90–100.

#### 3.2.3 Schizophrenia case study

The CMPA analysis on both GSE53987 and GSE21138 datasets was unable to reveal any relevant mechanisms in play. In GSE53987, only two biological processes, cell death (GO:0008219) and autophagy (GO:0006914), were notably downregulated. Conversely, an unexpected upregulation of Gamma Rhythm (MESH: D065818) appeared in GSE21138. The role of autophagy and cell death has been studied and linked to schizophrenia, and it is understood that a loss in autophagy predisposes to neurodegeneration and mood disorders (Schneider et al., 2016). Meanwhile, gamma rhythm has been found to be negatively correlated with schizophrenia (Metzner et al., 2022). This negative correlation aligns with our schizophrenia KG; thus, we hypothesize the observed gamma rhythm upregulation as a dataset-specific anomaly. Although the GSEA analysis of both datasets failed to reveal any significant pathway dysregulation (fdr <= 0.05), we did observe some trends in the pathways, such as, the downregulation of the “IL17 signaling pathway” (WP2112) and the downregulation of “Caspase-mediated cleavage of cytoskeletal proteins” (R-HSA-264870) pathway. The IL17 signaling pathway is an immune response pathway whose constituent protein, IL17, is considered a predictive biomarker in schizophrenia (Ouyang et al., 2022) as its gene is found to be upregulated in patients with schizophrenia (Ghasemi Noghabi et al., 2024). Similarly, the Caspase-mediated cleavage of cytoskeletal proteins constitutes proteins from the caspase family, such as CASP3, CASP4, CASP7, which 8 are identified to be upregulated in schizophrenia and are linked to apoptosis (Sahu et al., 2019). We hypothesize that this discrepancy is due to the non-significant GSEA results. Finally, the CLEP algorithm was able to identify and cluster the samples from the brains of patients with schizophrenia and the samples from the brains of the unaffected controls for both GSE53987 and GSE21138 (Figures 6A,B).

**FIGURE 6:**
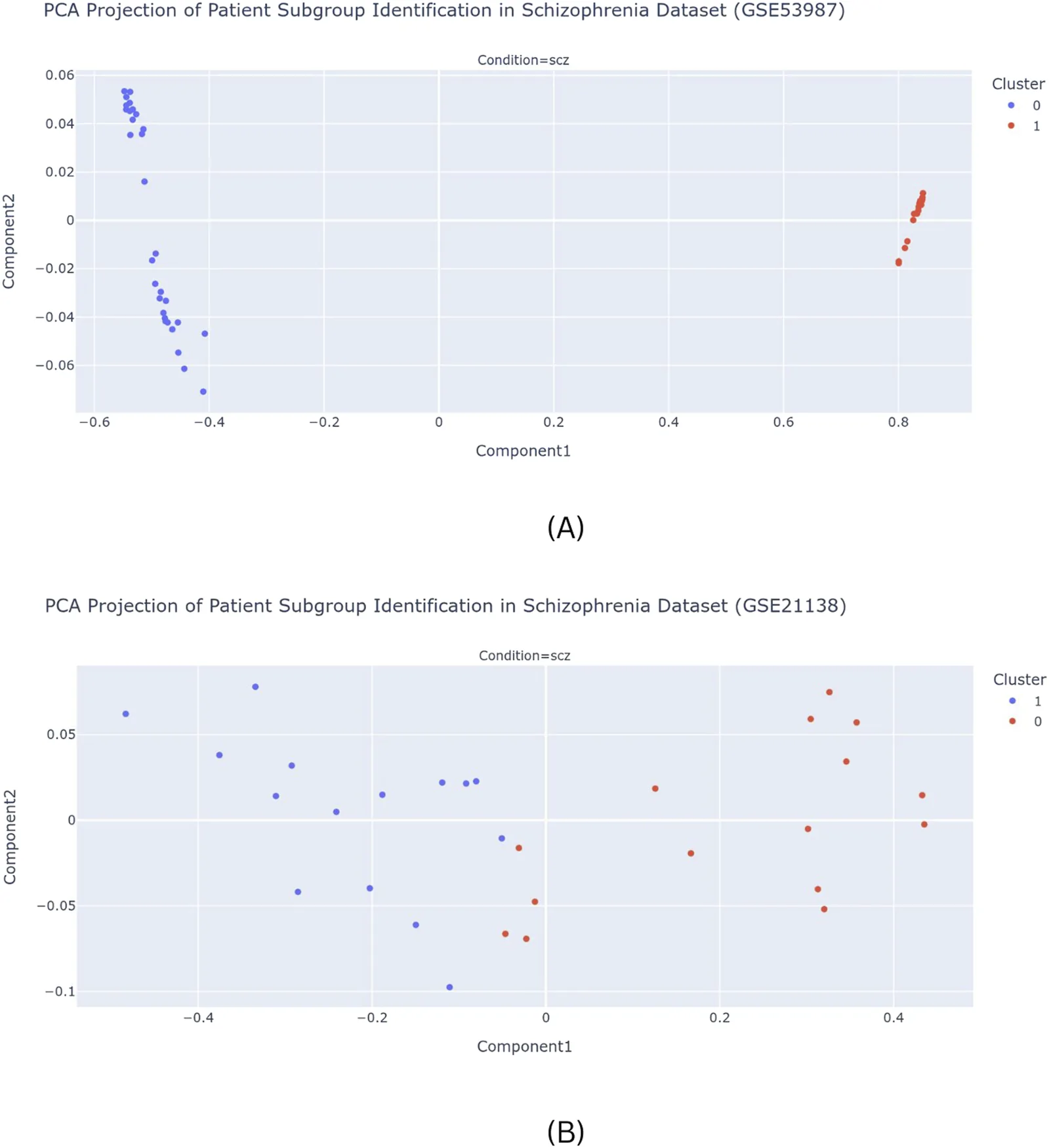
**(A)** CLEP Analysis of GSE53987. Clustering of the CLEP embeddings generated for GSE53987. **(B)** CLEP Analysis of GSE21138. Clustering of the CLEP embeddings generated for GSE21138.

## 4 Conclusion

We have developed a web-based research tool to support the exploration and analysis of neuropsychiatric and neurodegenerative disease mechanisms by integrating disease-specific KGs and multi-omics data. Rather than aiming to replace existing analysis workflows, the platform serves as a flexible framework for researchers to perform mechanism enrichment, patient stratification, and pathway analysis using established algorithms such as CMPA, CLEP, GSEA, and CARMA. Employment of multiple algorithms in the data analysis is key as shown in the ADNI case study as the results from different algorithms can aid in the result interpretation. Furthermore, the mechanistic and genomic results can be explored with the context of the disease KG using the explorative tool. This aids the users to generate a holistic hypothesis. A key feature of Neuro-PsychoMMSig is its support for self-hosted deployment. Researchers can deploy the platform locally, enabling secure, offline analysis of sensitive or proprietary datasets. This also allows users to incorporate custom KGs or extend the platform with new analysis algorithms tailored to their specific research needs. The modular architecture and use of widely adopted tools (e.g., Django, Neo4j, Docker) make the platform accessible and adaptable for a broad range of computational biology applications. The case studies presented here illustrate how the tool can be applied to generate mechanistic insights across diseases such as Alzheimer’s and schizophrenia. While the results are consistent with prior findings, they also highlight the importance of context-specific knowledge integration in interpreting high-dimensional biological data.

While the platform offers broad functionality, several limitations remain. Its current analyses rely on curated prior knowledge, and some methods, such as CLEP, are computationally intensive and may require dedicated hardware resources. Interpretation of stratification results, particularly in complex datasets, may also require further refinement. An integrated AI assistant would be very beneficial for Neuro-PsychoMMSig, enabling more interactive analysis for clinical researchers without a bioinformatics background. This improved usability will be used as a basis for future work where we will seek to expand interoperability further by incorporating more KG algorithms for neuroimaging, longitudinal clinical records and other diverse data types.

## Data Availability

The datasets analyzed in this publication can be found at Alzheimer’s Disease Neuroimaging Initiative (ADNI) database (adni.loni.usc.edu), and Gene expression Omnibus with the accession numbers, GSE33000, GSE53987 and GSE21138.

## Supplementary material

SUPPLEMENTARY TABLE 1 Workbook consisting of Knowledge graph node and edge attributes along with analysis results.

SUPPLEMENTARY IMAGE 1 Explorative analysis interface of Neuro-PsychoMMSig. The Knowledge Graphs in the web tool can be explored by selecting biomarkers of note and visualizing their interaction with other diseases or biomarkers.

SUPPLEMENTARY IMAGE 2A Explorative Analysis. The web UI for exploring the shared context between APOE in Alzheimer’s disease KG and NF-kappa β signalling pathway in schizophrenia KG.

SUPPLEMENTARY IMAGE 2B Visualization of explorative analysis. This is the resultant subgraph of the queried APOE node and NF-kappa β signalling pathway.

SUPPLEMENTARY IMAGE 3A The frontpage of Neuro-PsychoMMSig which features a quick search tool for visualizing the neighbourhood of biomarkers across all disease knowledge graphs.

SUPPLEMENTARY IMAGE 3B The Quick start guide for Neuro-PsychoMMSig to get started on analysis with clinical datasets.

SUPPLEMENTARY IMAGE 4 Clinical analysis results UI. Sample UI of the clinical analysis results using the GSEA algorithm.
